# The Phospha–Michael addition product {(*t*-BuNH)P(μ-N-*t*-Bu)_2_P(=N-*t*-Bu)—C(=CH_2_)CH(*p*-CH_3_O—C_6_H_4_)-P(O)[(OCH_2_C(CH_3_)_2_CH_2_O)]}

**DOI:** 10.1107/S1600536811014127

**Published:** 2011-04-29

**Authors:** G. Gangadhararao, Srinivas Venu

**Affiliations:** a School of Chemistry, University of Hyderabad, Hyderabad 500 046, India

## Abstract

The title compound, 2-{2-[1,3-di-*tert*-butyl-4-(*tert*-butyl­amino)-2-(*tert*-butyl­imino)-1,3,2λ^5^,4-diaza­diphosphetidin-2-yl]-1-(4-meth­oxy­phen­yl)prop-2-en-1-yl}-5,5-dimethyl-1,3,2λ^5^-dioxaphosphinan-2-one, C_31_H_57_N_4_O_4_P_3_, was synthesized from the Phospha–Michael addition reaction of cyclo­diphos­pha­zane [(*t*-BuNH)P(μ-N*t*-Bu)]_2_ and allenyl­phospho­nate [(OCH_2_C(CH_3_)_2_CH_2_O)P(O)C(*p*-CH_3_O—C_6_H_4_)=C=CH_2_]. In the crystal, N—H⋯O and C—H⋯O hydrogen bonds link the mol­ecules. The structure exhibits pseudosymmetry but attempts to solve it in a higher (monoclinic) space group were unsuccessful.

## Related literature

For background to cyclo­diphosph(III)aza­nes, see: Rama Suresh *et al.* (2009 ▶); Balakrishna (2010 ▶); Balakrishna *et al.* (2010 ▶). For their use as probes for organic reactions (the P atom reacts readily with activated alkenes/alkynes or azodicarboxyl­ates), see: Satish Kumar *et al.* (2004 ▶); Praveen Kumar *et al.* (2004 ▶); Balaraman & Kumara Swamy (2004 ▶); Bhuvan Kumar & Kumara Swamy (2007 ▶, 2008 ▶); Kumara Swamy *et al.* (2010 ▶, 2011 ▶). It has been shown recently that their reactions with allenes generates a chiral carbon center and in some cases spontaneous resolution by crystallization can be effected (Bhuvan Kumar & Kumara Swamy (2008 ▶). For related structures, see: Chakravarty *et al.* (2005 ▶); Kumara Swamy *et al.* (2010 ▶, 2011 ▶).
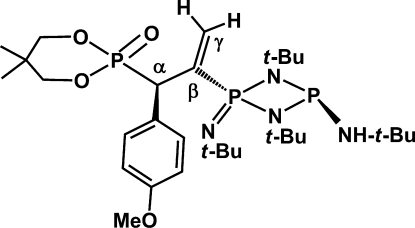


## Experimental

### 

#### Crystal data

C_31_H_57_N_4_O_4_P_3_
*M* *_r_* = 642.72
Triclinic, 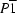

*a* = 13.8603 (9) Å
*b* = 15.7746 (10) Å
*c* = 16.2606 (11) Å
α = 88.004 (1)°
β = 84.949 (1)°
γ = 87.600 (1)°
*V* = 3536.6 (4) Å^3^
*Z* = 4
Mo *K*α radiation
μ = 0.21 mm^−1^
*T* = 100 K
0.22 × 0.18 × 0.14 mm

#### Data collection

Bruker SMART CCD area-detector diffractometer
Absorption correction: multi-scan (*SADABS*; Sheldrick, 1996 ▶) *T*_min_ = 0.956, *T*_max_ = 0.972
27730 measured reflections
12397 independent reflections
9894 reflections with *I* > 2σ(*I*)
*R*_int_ = 0.033

#### Refinement

*R*[*F* ^2^ > 2σ(*F* ^2^)] = 0.049
*wR*(*F* ^2^) = 0.128
*S* = 1.01
12397 reflections
795 parameters
H atoms treated by a mixture of independent and constrained refinement
Δρ_max_ = 0.54 e Å^−3^
Δρ_min_ = −0.28 e Å^−3^

### 

Data collection: *SMART* (Bruker, 2002 ▶); cell refinement: *SAINT* (Bruker, 2002 ▶); data reduction: *SAINT*; program(s) used to solve structure: *SHELXS97* (Sheldrick, 2008 ▶); program(s) used to refine structure: *SHELXL97* (Sheldrick, 2008 ▶); molecular graphics: *SHELXTL* (Sheldrick, 2008 ▶); software used to prepare material for publication: *SHELXTL*.

## Supplementary Material

Crystal structure: contains datablocks I, global. DOI: 10.1107/S1600536811014127/zj2002sup1.cif

Structure factors: contains datablocks I. DOI: 10.1107/S1600536811014127/zj2002Isup2.hkl

Additional supplementary materials:  crystallographic information; 3D view; checkCIF report

## Figures and Tables

**Table 1:** Hydrogen-bond geometry (Å, °)

*D*—H⋯*A*	*D*—H	H⋯*A*	*D*⋯*A*	*D*—H⋯*A*
N8—H8*D*⋯O1^i^	0.82 (2)	2.61 (3)	3.394 (2)	160 (2)
N4—H4*D*⋯O5^ii^	0.84 (3)	2.58 (3)	3.378 (2)	158 (2)
C26—H26*B*⋯O5^iii^	0.96	2.49	3.325 (3)	145

Symmetry codes: (i) 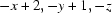; (ii) 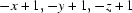; (iii) 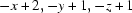.

**Fig. 1.**
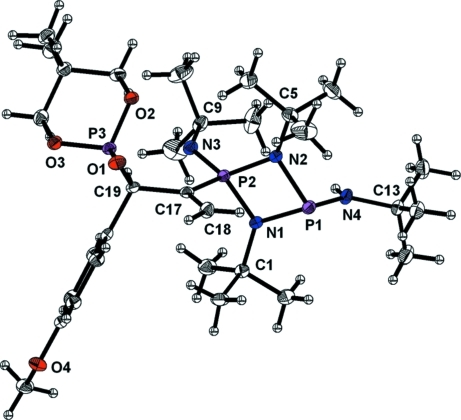
An ORTEP representation of compound 1 (Thermal ellipsoids are at 50% probability level). Two molecules are present in the asymmetric unit. Second molecule is not shown for clarity.

**Fig. 2.**
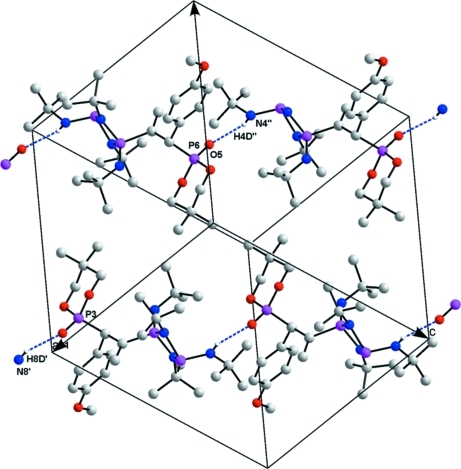
1-Dimensional structural unit due to the N—H···O interactions in the crystal structure of compound 1.

## supplementary crystallographic information

### Comment

Cyclodiphosph(III)azanes are good phosphorus based ligands (Rama Suresh *et al.*, 2009; Balakrishna *et al.*, 2010). They can also act as probes for organic reactions since the P(III) centre reacts readily with activated alkenes/ alkynes or azodicarboxylates (Satish Kumar *et al.*, 2004; Praveen Kumar *et al.*, 2004; Balaraman *et al.*, 2004; Bhuvan Kumar *et al.*, 2007; 2008; Kumara Swamy *et al.*, 2010; 2011). It has been shown recently that their reactions with allenes generates a chiral carbon center and in some cases spontaneous resolution by crystallization can be effected (Bhuvan Kumar *et al.*, 2008). To probe this aspect further we reacted cyclodiphosph(III)azane [(*t*-BuNH)P(µ-N-*t*-Bu)]_2_ (1a) with allene (OCH_2_CMe_2_CH_2_O)P(O)C(C_6_H_4_-*p*-OCH_3_)═C═CH_2_ (1b). The reaction afforded compound 1 (Scheme 1) as essentially a single product. The X-ray structure of compound 1 [Figure 1] clearly show the phosphinimine moiety at P(2) and P(5) with P—N distances of 1.546 (4) and 1.546 (1) that are slightly longer than the structures reported earlier (Chakravarty *et al.*, 2005; Kumara Swamy *et al.*, 2010). However, these are still in the range expected for P=N distances; the P—N single bond distances in the cyclophosphazane ring also lie in the same range of the previously reported structures (Kumara Swamy *et al.*, 2011). However, the P—N(ring) distances involving P(III) phosphorus atoms are comparatively longer than that for P(V) phosphorus. The P–C distances are also in line with the structures as written. The P—N—P and N—P—N bond angles are also in the expected range.The crystal packing in compound 1 is mostly governed by classical hydrogen bonds. The two molecules in the asymmetric unit are extended in a one-dimensional fashion through N—H···O interactions [N(4)—H(4D)···O5 and N(8)—H(8D)···O(1)] (Figure 2). But only the first molecule was also having C—H···O(=P) [C26—H(26B)···O(5)] interactions by OC*H*_3_ hydrogen and with the phosphoryl oxygen (P═*O*) of the second molecule. The crystal structure is showing an alert due to the psuedo symmetry. We have investigated the possibility of solving in higher symmetry space group and also the presence of pseudo-centre of symmetry. All our attempts to solve the structure in a higher symmetry (in Monoclinic) space group were not successful. Therefore, we strongly believe that there is only a pseudo-symmetry.

### Experimental

Comopound 1: This compound (1) was obtained by the reaction of cyclodiphosphazane 1a (0.561 g, 1.61 mmol) and allenylphosphonate 1b (0 474 g, 1.61 mmol) in dry toluene (8 ml) for 20 h. The solution (toluene) was concentrated *in vacuo* (to *ca* 3 ml) and cooled for 1 day at -4°C to obtain the colorless crystals of product. Yield: 0.956 g (92%). mp: 150–154°C. IR (KBr, cm^-1^): 3337, 2967, 2897, 1615, 1584, 1510, 1464, 1364, 1281, 1209, 1063, 1028, 885. ^1^H NMR (400 MHz, CDCl_3_): δ 0.83, 1.08, 1.18, 1.29 and 1.37 (5 s, 42H, C(C*H*_3_)_2_+C(C*H*_3_)_3_), 2.69 (d, ^2^*J*(P—H) = 7.6 Hz, 1H, N*H*), 3.78 (s, 3H, Ar—OC*H*_3_) 3.82–4.36 (m, 4H, OC*H*_2_), 5.76 (d, ^3^*J*(P—H) = 28.8 Hz, 1H, =C*H*_A_H_B_*cis* to P), 6.00 (dd, ^3^*J*(P—H) = 15.2 Hz, ^2^*J*(P—H) = 18.0 Hz, 1H, P(O)C*H*), 6.76 (d, ^3^*J*(P—H) = 52.4 Hz, 1H, =CH_A_*H*_B_*trans* to P), 6.81 (d, ^3^*J*(H—H) = 8.4 Hz, 2H, Ar-*H)*, 7.47 (d, ^3^*J*(H—H) = 8.0 Hz, 2H, Ar-*H*). ^13^C NMR (100 MHz, CDCl_3_): δ 20.81 and 21.85 (2 s, C(*C*H_3_)_2_), 31.10 (d, ^3^*J*(P—C) = 23.4 Hz, C(*C*H_3_)_3_), 32.31 (d, ^3^*J*(P—C) = 6.0 Hz, C(*C*H_3_)_3_), 32.78 (d, ^3^*J*(P—C) = 9.2 Hz, C(*C*H_3_)_3_), 34.39 (d, ^3^*J*(P—C) = 11.2 Hz, C(*C*H_3_)_3_), 40.72 (d, ^1^*J*(P—C) = 125.4 Hz, P(O)*C*(Ar)), 51.31 (d, ^2^*J*(P—C) = 14.5 Hz, *C*(CH_3_)_3_), 52.25 (d, ^2^*J*(P—C) = 8.8 Hz, *C*(CH_3_)_3_), 52.51 (d, ^2^*J*(P—C) = 7.7 Hz, *C*(CH_3_)_3_), 55.08 (Ar—O*C*H_3_),76.38 and 76.42 (2 s, O*C*H_2_), 113.52, 128.06, 131.05, 131.12, 158.67 (d, ^2^*J*(P—C) = 6.4 Hz, Ar-*C* + PC=*C*H_2_), 143.56 (d, ^1^*J*(P—C) = 159.0 Hz, P*C*=CH_2_). ^31^P NMR (160 MHz, CDCl_3_): δ -18.90 (dd, ^3^*J*(P—P) = 35.8 Hz, ^2^*J*(P—P) = 6.7 Hz), 20.91 (d, ^3^*J*(P—P) = 35.8 Hz), 70.99 (br d, ^2^*J*(P—P) = 6.7 Hz). LC—MS: m/*z* 643 [*M*+1]^+^. Anal. Calc. For C_31_H_57_N_4_O_4_P_3_: C, 57.93; H, 8.94; N, 8.72. Found: C, 57.79; H, 8.98; N, 8.82. A suitable crystal (Shape: blocks: Size: 0.22 *x* 0.18 *x* 0.14 mm^3^) was mounted on a glass fibre and data was collected on BRUKER *SMART* diffractometer at 100 K.

### Refinement

All H atoms were found on difference maps, with C—H=0.93 Å and included in the final cycles of refinement using a riding model, with *U*_iso_(H)=1.2Ueq(C)

The structure was solved by direct methods and refined by full-matrix least squares methods using standard procedures. Absorption corrections were done using the *SADABS* program, where applicable. All non-hydrogen atoms were refined anisotropically; hydrogen atoms were fixed by geometry or located by a difference Fourier and refined isotropically.

### Crystal data

**Table:**

C_31_H_57_N_4_O_4_P_3_	*Z* = 4
*M**_r_* = 642.72	*F*(000) = 1392
Triclinic, *P*1	*D*_x_ = 1.207 Mg m^−^^3^
Hall symbol: -P 1	Mo *K*α radiation, λ = 0.71073 Å
*a* = 13.8603 (9) Å	Cell parameters from 6427 reflections
*b* = 15.7746 (10) Å	θ = 2.4–26.0°
*c* = 16.2606 (11) Å	µ = 0.21 mm^−^^1^
α = 88.004 (1)°	*T* = 100 K
β = 84.949 (1)°	Blocks, colorless
γ = 87.600 (1)°	0.22 × 0.18 × 0.14 mm
*V* = 3536.6 (4) Å^3^	

### Data collection

**Table:**

Bruker SMART CCD area-detector diffractometer	12397 independent reflections
Radiation source: fine-focus sealed tube	9894 reflections with *I* > 2σ(*I*)
graphite	*R*_int_ = 0.033
φ and ω scans	θ_max_ = 25.0°, θ_min_ = 1.3°
Absorption correction: multi-scan (*SADABS*; Sheldrick, 1996)	*h* = −16→16
*T*_min_ = 0.956, *T*_max_ = 0.972	*k* = −18→18
27730 measured reflections	*l* = −19→19

### Refinement

**Table:**

Refinement on *F*^2^	Primary atom site location: structure-invariant direct methods
Least-squares matrix: full	Secondary atom site location: difference Fourier map
*R*[*F*^2^ > 2σ(*F*^2^)] = 0.049	Hydrogen site location: inferred from neighbouring sites
*wR*(*F*^2^) = 0.128	H atoms treated by a mixture of independent and constrained refinement
*S* = 1.01	*w* = 1/[σ^2^(*F*_o_^2^) + (0.0649*P*)^2^ + 1.2977*P*] where *P* = (*F*_o_^2^ + 2*F*_c_^2^)/3
12397 reflections	(Δ/σ)_max_ = 0.001
795 parameters	Δρ_max_ = 0.54 e Å^−^^3^
0 restraints	Δρ_min_ = −0.28 e Å^−^^3^

### Special details

**Table:**

Experimental. A colorless block with approximate orthogonal dimensions 0.22 *x* 0.18 *x* 0.14 mm3 was placed and optically centered on the Bruker *SMART* CCD system at 100 (2) K.
Geometry. All e.s.d.'s (except the e.s.d. in the dihedral angle between two l.s. planes) are estimated using the full covariance matrix. The cell e.s.d.'s are taken into account individually in the estimation of e.s.d.'s in distances, angles and torsion angles; correlations between e.s.d.'s in cell parameters are only used when they are defined by crystal symmetry. An approximate (isotropic) treatment of cell e.s.d.'s is used for estimating e.s.d.'s involving l.s. planes.
Refinement. Refinement of *F*^2^ against ALL reflections. The weighted *R*-factor *wR* and goodness of fit *S* are based on *F*^2^, conventional *R*-factors *R* are based on *F*, with *F* set to zero for negative *F*^2^. The threshold expression of *F*^2^ > σ(*F*^2^) is used only for calculating *R*-factors(gt) *etc*. and is not relevant to the choice of reflections for refinement. *R*-factors based on *F*^2^ are statistically about twice as large as those based on *F*, and *R*- factors based on ALL data will be even larger.

### Fractional atomic coordinates and isotropic or equivalent isotropic displacement parameters (Å^2^)

**Table:**

	*x*	*y*	*z*	*U*_iso_*/*U*_eq_	
P5	0.59309 (4)	0.74569 (3)	0.08104 (3)	0.01270 (13)	
P6	0.40564 (4)	0.66855 (3)	0.27677 (3)	0.01388 (13)	
P4	0.66984 (4)	0.88814 (3)	0.03551 (3)	0.01523 (14)	
O5	0.37256 (11)	0.74031 (9)	0.32774 (9)	0.0187 (3)	
O8	0.03668 (11)	0.75573 (10)	0.05412 (9)	0.0233 (4)	
O6	0.33881 (10)	0.58979 (9)	0.29393 (9)	0.0169 (3)	
O7	0.50990 (10)	0.63320 (9)	0.29590 (9)	0.0157 (3)	
N6	0.67820 (12)	0.80751 (11)	0.11155 (10)	0.0148 (4)	
C54	0.12697 (15)	0.73681 (13)	0.07809 (13)	0.0170 (5)	
C48	0.47736 (15)	0.76502 (13)	0.14219 (12)	0.0137 (4)	
C55	0.14671 (15)	0.76903 (13)	0.15315 (13)	0.0168 (5)	
H55	0.0990	0.8006	0.1837	0.020*	
N7	0.60389 (12)	0.64798 (11)	0.07626 (10)	0.0155 (4)	
C51	0.30881 (15)	0.70578 (13)	0.13897 (12)	0.0143 (4)	
N8	0.77127 (13)	0.88560 (12)	−0.02712 (12)	0.0186 (4)	
C56	0.23648 (15)	0.75457 (13)	0.18261 (13)	0.0173 (5)	
H56	0.2490	0.7776	0.2323	0.021*	
C58	0.54361 (15)	0.55016 (13)	0.26565 (13)	0.0175 (5)	
H58A	0.6067	0.5355	0.2849	0.021*	
H58B	0.5507	0.5526	0.2057	0.021*	
C35	0.45625 (16)	0.76226 (15)	−0.06387 (14)	0.0235 (5)	
H35A	0.4821	0.7054	−0.0563	0.035*	
H35B	0.4217	0.7659	−0.1126	0.035*	
H35C	0.4128	0.7768	−0.0168	0.035*	
C52	0.28674 (15)	0.67198 (13)	0.06574 (13)	0.0151 (5)	
H52	0.3332	0.6377	0.0367	0.018*	
C36	0.72204 (16)	0.81403 (14)	0.19125 (13)	0.0187 (5)	
C49	0.45156 (16)	0.84466 (13)	0.16018 (13)	0.0193 (5)	
H49A	0.3916	0.8571	0.1884	0.023*	
H49B	0.4934	0.8881	0.1446	0.023*	
C57	0.01704 (17)	0.72673 (15)	−0.02482 (14)	0.0245 (5)	
H57A	0.0615	0.7511	−0.0668	0.037*	
H57B	−0.0482	0.7436	−0.0355	0.037*	
H57C	0.0246	0.6660	−0.0252	0.037*	
C53	0.19755 (15)	0.68757 (13)	0.03410 (13)	0.0171 (5)	
H53	0.1853	0.6653	−0.0160	0.020*	
C32	0.53851 (15)	0.82327 (13)	−0.07322 (13)	0.0165 (5)	
C40	0.68215 (16)	0.59654 (13)	0.03178 (13)	0.0176 (5)	
C59	0.47325 (16)	0.48183 (13)	0.29526 (13)	0.0189 (5)	
N5	0.59713 (12)	0.81344 (11)	−0.00120 (10)	0.0148 (4)	
C50	0.41072 (15)	0.69154 (13)	0.16671 (12)	0.0140 (4)	
H50	0.4396	0.6411	0.1392	0.017*	
C34	0.49557 (18)	0.91388 (14)	−0.07822 (15)	0.0268 (6)	
H34A	0.4531	0.9245	−0.0295	0.040*	
H34B	0.4597	0.9209	−0.1261	0.040*	
H34C	0.5469	0.9532	−0.0822	0.040*	
C60	0.37424 (16)	0.50669 (13)	0.26594 (14)	0.0205 (5)	
H60A	0.3786	0.5070	0.2061	0.025*	
H60B	0.3283	0.4646	0.2864	0.025*	
C33	0.60321 (17)	0.80532 (15)	−0.15207 (13)	0.0237 (5)	
H33A	0.6539	0.8453	−0.1586	0.036*	
H33B	0.5652	0.8107	−0.1987	0.036*	
H33C	0.6312	0.7488	−0.1485	0.036*	
C61	0.50832 (18)	0.39855 (14)	0.25481 (15)	0.0277 (6)	
H61A	0.5107	0.4061	0.1958	0.041*	
H61B	0.4644	0.3548	0.2726	0.041*	
H61C	0.5719	0.3826	0.2706	0.041*	
C44	0.82293 (16)	0.96108 (13)	−0.06183 (14)	0.0189 (5)	
C45	0.78670 (18)	0.98684 (15)	−0.14567 (15)	0.0296 (6)	
H45A	0.7964	0.9399	−0.1819	0.044*	
H45B	0.8221	1.0341	−0.1692	0.044*	
H45C	0.7189	1.0027	−0.1385	0.044*	
C43	0.70774 (17)	0.52019 (14)	0.08743 (14)	0.0247 (5)	
H43A	0.7288	0.5397	0.1379	0.037*	
H43B	0.7589	0.4862	0.0597	0.037*	
H43C	0.6517	0.4867	0.0996	0.037*	
C37	0.82582 (18)	0.84214 (18)	0.17242 (16)	0.0358 (7)	
H37A	0.8616	0.8018	0.1375	0.054*	
H37B	0.8560	0.8454	0.2231	0.054*	
H37C	0.8252	0.8969	0.1448	0.054*	
C39	0.72334 (17)	0.72728 (14)	0.23515 (14)	0.0246 (5)	
H39A	0.6581	0.7093	0.2470	0.037*	
H39B	0.7534	0.7308	0.2859	0.037*	
H39C	0.7594	0.6870	0.2004	0.037*	
C62	0.46740 (17)	0.47035 (14)	0.38906 (13)	0.0224 (5)	
H62A	0.5285	0.4478	0.4054	0.034*	
H62B	0.4175	0.4318	0.4069	0.034*	
H62C	0.4526	0.5242	0.4138	0.034*	
C41	0.64407 (17)	0.56365 (14)	−0.04624 (13)	0.0225 (5)	
H41A	0.5857	0.5339	−0.0315	0.034*	
H41B	0.6920	0.5258	−0.0730	0.034*	
H41C	0.6306	0.6106	−0.0832	0.034*	
C38	0.6647 (2)	0.87766 (16)	0.24708 (15)	0.0341 (6)	
H38A	0.6593	0.9315	0.2180	0.051*	
H38B	0.6976	0.8843	0.2958	0.051*	
H38C	0.6011	0.8573	0.2624	0.051*	
C42	0.77415 (16)	0.64415 (14)	0.00713 (14)	0.0232 (5)	
H42A	0.7601	0.6900	−0.0309	0.035*	
H42B	0.8225	0.6060	−0.0187	0.035*	
H42C	0.7978	0.6664	0.0554	0.035*	
C47	0.80763 (18)	1.03499 (14)	−0.00409 (15)	0.0288 (6)	
H47A	0.7402	1.0522	0.0015	0.043*	
H47B	0.8450	1.0817	−0.0264	0.043*	
H47C	0.8281	1.0178	0.0491	0.043*	
C46	0.93066 (16)	0.93594 (15)	−0.07427 (15)	0.0258 (5)	
H46A	0.9538	0.9188	−0.0221	0.039*	
H46B	0.9659	0.9835	−0.0973	0.039*	
H46C	0.9400	0.8896	−0.1113	0.039*	
P2	0.89633 (4)	0.24677 (3)	0.41973 (3)	0.01371 (13)	
P1	0.82093 (4)	0.10583 (3)	0.47234 (3)	0.01574 (14)	
P3	1.09056 (4)	0.32194 (3)	0.22058 (3)	0.01479 (14)	
O4	1.43525 (11)	0.25060 (10)	0.47038 (9)	0.0215 (4)	
O2	0.98715 (10)	0.35323 (9)	0.19487 (9)	0.0164 (3)	
O1	1.13188 (11)	0.25206 (9)	0.17058 (9)	0.0196 (3)	
O3	1.15386 (10)	0.40346 (9)	0.20836 (9)	0.0179 (3)	
C23	1.34641 (15)	0.26494 (13)	0.44149 (13)	0.0164 (5)	
C21	1.18557 (15)	0.32641 (13)	0.44112 (13)	0.0162 (5)	
H21	1.1360	0.3610	0.4657	0.019*	
N1	0.89654 (12)	0.18141 (11)	0.50433 (10)	0.0151 (4)	
C25	1.24771 (15)	0.23654 (13)	0.33221 (13)	0.0167 (5)	
H25	1.2400	0.2092	0.2836	0.020*	
C18	1.02663 (16)	0.14940 (13)	0.32540 (13)	0.0197 (5)	
H18A	1.0838	0.1376	0.2928	0.024*	
H18B	0.9835	0.1066	0.3394	0.024*	
C17	1.00657 (15)	0.22708 (13)	0.35183 (12)	0.0143 (4)	
N3	0.88579 (13)	0.34459 (11)	0.42252 (11)	0.0172 (4)	
C19	1.07475 (15)	0.30000 (13)	0.33072 (12)	0.0139 (4)	
H19	1.0426	0.3508	0.3553	0.017*	
N2	0.81034 (12)	0.18369 (11)	0.39409 (11)	0.0157 (4)	
C28	1.01401 (16)	0.50577 (13)	0.20302 (13)	0.0184 (5)	
C24	1.33385 (15)	0.22616 (13)	0.36802 (13)	0.0171 (5)	
H24	1.3842	0.1928	0.3428	0.020*	
C20	1.17163 (15)	0.28768 (13)	0.36797 (13)	0.0148 (4)	
C26	1.45102 (17)	0.28712 (14)	0.54678 (13)	0.0231 (5)	
H26A	1.4407	0.3476	0.5423	0.035*	
H26B	1.5163	0.2739	0.5596	0.035*	
H26C	1.4067	0.2644	0.5898	0.035*	
N4	0.72237 (13)	0.11378 (12)	0.53761 (11)	0.0174 (4)	
C29	0.94605 (16)	0.43378 (13)	0.22661 (13)	0.0182 (5)	
H29A	0.9348	0.4289	0.2863	0.022*	
H29B	0.8842	0.4465	0.2043	0.022*	
C5	0.75761 (16)	0.17757 (14)	0.31903 (14)	0.0219 (5)	
C27	1.11183 (16)	0.48426 (13)	0.23657 (14)	0.0204 (5)	
H27A	1.1561	0.5287	0.2192	0.024*	
H27B	1.1036	0.4825	0.2965	0.024*	
C13	0.66233 (16)	0.04273 (13)	0.57061 (14)	0.0189 (5)	
C4	0.98429 (18)	0.25201 (14)	0.60522 (14)	0.0261 (5)	
H4A	1.0054	0.2922	0.5624	0.039*	
H4B	1.0331	0.2441	0.6435	0.039*	
H4C	0.9250	0.2730	0.6338	0.039*	
C2	0.92494 (17)	0.10746 (15)	0.63526 (14)	0.0249 (5)	
H2A	0.8647	0.1316	0.6594	0.037*	
H2B	0.9695	0.0990	0.6770	0.037*	
H2C	0.9138	0.0539	0.6121	0.037*	
C3	1.06421 (16)	0.12822 (15)	0.53081 (14)	0.0237 (5)	
H3A	1.0527	0.0767	0.5042	0.036*	
H3B	1.1058	0.1159	0.5742	0.036*	
H3C	1.0947	0.1674	0.4912	0.036*	
C7	0.64933 (17)	0.17330 (18)	0.34434 (16)	0.0352 (6)	
H7A	0.6375	0.1238	0.3793	0.053*	
H7B	0.6149	0.1702	0.2959	0.053*	
H7C	0.6274	0.2232	0.3738	0.053*	
C30	0.97184 (18)	0.58638 (14)	0.24454 (15)	0.0284 (6)	
H30A	0.9085	0.5997	0.2270	0.043*	
H30B	1.0133	0.6326	0.2293	0.043*	
H30C	0.9673	0.5774	0.3034	0.043*	
C16	0.67254 (17)	−0.03126 (14)	0.51240 (14)	0.0237 (5)	
H16A	0.6572	−0.0117	0.4583	0.036*	
H16B	0.6289	−0.0745	0.5326	0.036*	
H16C	0.7379	−0.0542	0.5095	0.036*	
C9	0.80892 (16)	0.39872 (13)	0.46543 (13)	0.0195 (5)	
C1	0.96760 (16)	0.16767 (13)	0.56725 (13)	0.0177 (5)	
C31	1.02493 (17)	0.51997 (14)	0.10952 (13)	0.0229 (5)	
H31A	1.0457	0.4676	0.0838	0.034*	
H31B	1.0722	0.5619	0.0950	0.034*	
H31C	0.9638	0.5393	0.0908	0.034*	
C14	0.55715 (16)	0.07624 (15)	0.57986 (16)	0.0283 (6)	
H14A	0.5510	0.1221	0.6174	0.043*	
H14B	0.5161	0.0315	0.6010	0.043*	
H14C	0.5382	0.0962	0.5270	0.043*	
C15	0.69229 (18)	0.01271 (15)	0.65535 (14)	0.0283 (6)	
H15A	0.7562	−0.0135	0.6493	0.042*	
H15B	0.6473	−0.0276	0.6796	0.042*	
H15C	0.6922	0.0604	0.6904	0.042*	
C12	0.7760 (2)	0.46657 (16)	0.40394 (16)	0.0368 (7)	
H12A	0.8295	0.5010	0.3852	0.055*	
H12B	0.7247	0.5015	0.4302	0.055*	
H12C	0.7529	0.4399	0.3576	0.055*	
C6	0.77596 (19)	0.25584 (16)	0.26283 (15)	0.0331 (6)	
H6A	0.7556	0.3060	0.2924	0.050*	
H6B	0.7399	0.2530	0.2153	0.050*	
H6C	0.8439	0.2577	0.2454	0.050*	
C11	0.8506 (2)	0.44271 (16)	0.53541 (17)	0.0392 (7)	
H11A	0.8656	0.4014	0.5777	0.059*	
H11B	0.8039	0.4841	0.5582	0.059*	
H11C	0.9085	0.4703	0.5146	0.059*	
C10	0.72105 (18)	0.35083 (15)	0.49979 (17)	0.0358 (7)	
H10A	0.6933	0.3244	0.4556	0.054*	
H10B	0.6740	0.3896	0.5263	0.054*	
H10C	0.7402	0.3081	0.5393	0.054*	
C8	0.7922 (2)	0.09845 (17)	0.27171 (17)	0.0404 (7)	
H8A	0.8581	0.1045	0.2494	0.061*	
H8B	0.7519	0.0916	0.2275	0.061*	
H8C	0.7883	0.0495	0.3085	0.061*	
C22	1.27127 (16)	0.31485 (13)	0.47850 (13)	0.0182 (5)	
H22	1.2784	0.3404	0.5282	0.022*	
H4D	0.7089 (18)	0.1594 (17)	0.5623 (16)	0.032 (7)*	
H8D	0.7859 (18)	0.8433 (16)	−0.0543 (15)	0.026 (7)*	

### Atomic displacement parameters (Å^2^)

**Table:**

	*U*^11^	*U*^22^	*U*^33^	*U*^12^	*U*^13^	*U*^23^
P5	0.0127 (3)	0.0133 (3)	0.0118 (3)	0.0010 (2)	−0.0003 (2)	0.0008 (2)
P6	0.0142 (3)	0.0154 (3)	0.0120 (3)	−0.0005 (2)	−0.0021 (2)	0.0019 (2)
P4	0.0160 (3)	0.0137 (3)	0.0158 (3)	0.0000 (2)	−0.0008 (2)	0.0016 (2)
O5	0.0221 (8)	0.0195 (8)	0.0140 (8)	0.0026 (6)	−0.0009 (6)	−0.0003 (6)
O8	0.0168 (8)	0.0291 (9)	0.0250 (9)	0.0032 (7)	−0.0088 (7)	−0.0021 (7)
O6	0.0161 (8)	0.0170 (8)	0.0171 (8)	−0.0010 (6)	−0.0005 (6)	0.0034 (6)
O7	0.0162 (8)	0.0151 (8)	0.0162 (8)	−0.0006 (6)	−0.0040 (6)	0.0021 (6)
N6	0.0150 (9)	0.0154 (9)	0.0140 (9)	−0.0006 (7)	−0.0026 (7)	0.0019 (7)
C54	0.0142 (11)	0.0157 (11)	0.0216 (12)	−0.0008 (9)	−0.0067 (9)	0.0056 (9)
C48	0.0144 (11)	0.0163 (11)	0.0106 (10)	0.0003 (9)	−0.0031 (9)	0.0021 (8)
C55	0.0170 (11)	0.0143 (11)	0.0186 (11)	0.0015 (9)	0.0004 (9)	−0.0002 (9)
N7	0.0150 (9)	0.0159 (9)	0.0149 (9)	0.0027 (7)	0.0000 (7)	−0.0002 (7)
C51	0.0157 (11)	0.0124 (10)	0.0147 (11)	−0.0011 (8)	−0.0017 (9)	0.0024 (8)
N8	0.0208 (10)	0.0127 (10)	0.0215 (10)	−0.0016 (8)	0.0022 (8)	0.0007 (8)
C56	0.0183 (12)	0.0174 (11)	0.0163 (11)	−0.0002 (9)	−0.0034 (9)	−0.0009 (9)
C58	0.0170 (11)	0.0166 (11)	0.0185 (11)	0.0034 (9)	−0.0023 (9)	0.0014 (9)
C35	0.0223 (13)	0.0314 (13)	0.0176 (12)	−0.0053 (10)	−0.0072 (10)	0.0041 (10)
C52	0.0161 (11)	0.0127 (10)	0.0161 (11)	0.0000 (9)	0.0010 (9)	0.0003 (8)
C36	0.0176 (12)	0.0229 (12)	0.0167 (11)	−0.0027 (9)	−0.0058 (9)	−0.0003 (9)
C49	0.0176 (12)	0.0172 (11)	0.0219 (12)	−0.0003 (9)	0.0042 (10)	0.0016 (9)
C57	0.0242 (13)	0.0259 (13)	0.0254 (13)	−0.0018 (10)	−0.0137 (10)	0.0034 (10)
C53	0.0217 (12)	0.0165 (11)	0.0138 (11)	−0.0030 (9)	−0.0047 (9)	0.0001 (9)
C32	0.0180 (11)	0.0177 (11)	0.0144 (11)	0.0013 (9)	−0.0062 (9)	0.0017 (9)
C40	0.0190 (12)	0.0145 (11)	0.0186 (11)	0.0038 (9)	0.0014 (9)	−0.0014 (9)
C59	0.0209 (12)	0.0165 (11)	0.0190 (12)	0.0010 (9)	−0.0017 (10)	0.0014 (9)
N5	0.0155 (9)	0.0150 (9)	0.0137 (9)	0.0004 (7)	−0.0012 (7)	0.0023 (7)
C50	0.0155 (11)	0.0132 (10)	0.0129 (10)	0.0019 (8)	−0.0010 (9)	0.0011 (8)
C34	0.0314 (14)	0.0239 (13)	0.0262 (13)	0.0060 (11)	−0.0131 (11)	0.0020 (10)
C60	0.0228 (12)	0.0167 (11)	0.0222 (12)	−0.0023 (9)	−0.0024 (10)	0.0002 (9)
C33	0.0254 (13)	0.0301 (13)	0.0157 (12)	−0.0033 (10)	−0.0021 (10)	0.0006 (10)
C61	0.0319 (14)	0.0208 (12)	0.0295 (14)	0.0028 (11)	0.0000 (11)	−0.0009 (10)
C44	0.0197 (12)	0.0152 (11)	0.0212 (12)	−0.0033 (9)	0.0005 (10)	0.0036 (9)
C45	0.0341 (15)	0.0248 (13)	0.0300 (14)	−0.0074 (11)	−0.0055 (11)	0.0116 (11)
C43	0.0268 (13)	0.0214 (12)	0.0235 (13)	0.0098 (10)	0.0044 (10)	0.0031 (10)
C37	0.0285 (14)	0.0532 (17)	0.0280 (14)	−0.0183 (13)	−0.0114 (12)	0.0102 (12)
C39	0.0281 (13)	0.0275 (13)	0.0192 (12)	−0.0042 (10)	−0.0080 (10)	0.0042 (10)
C62	0.0241 (13)	0.0230 (12)	0.0192 (12)	0.0004 (10)	−0.0014 (10)	0.0083 (9)
C41	0.0273 (13)	0.0201 (12)	0.0198 (12)	0.0034 (10)	−0.0017 (10)	−0.0036 (9)
C38	0.0461 (17)	0.0356 (15)	0.0225 (13)	0.0088 (12)	−0.0141 (12)	−0.0109 (11)
C42	0.0222 (12)	0.0216 (12)	0.0245 (13)	0.0015 (10)	0.0052 (10)	−0.0027 (10)
C47	0.0368 (15)	0.0197 (12)	0.0289 (14)	−0.0062 (11)	0.0047 (11)	0.0005 (10)
C46	0.0207 (13)	0.0245 (13)	0.0317 (14)	−0.0057 (10)	0.0022 (11)	0.0020 (10)
P2	0.0136 (3)	0.0131 (3)	0.0141 (3)	0.0003 (2)	−0.0009 (2)	0.0017 (2)
P1	0.0163 (3)	0.0140 (3)	0.0167 (3)	−0.0004 (2)	−0.0009 (2)	0.0017 (2)
P3	0.0148 (3)	0.0160 (3)	0.0133 (3)	0.0004 (2)	−0.0014 (2)	0.0017 (2)
O4	0.0194 (8)	0.0262 (9)	0.0199 (8)	0.0012 (7)	−0.0079 (7)	−0.0022 (7)
O2	0.0166 (8)	0.0162 (8)	0.0162 (8)	0.0009 (6)	−0.0021 (6)	0.0020 (6)
O1	0.0233 (9)	0.0194 (8)	0.0155 (8)	0.0040 (7)	−0.0010 (7)	−0.0004 (6)
O3	0.0169 (8)	0.0181 (8)	0.0182 (8)	0.0005 (6)	−0.0002 (6)	0.0029 (6)
C23	0.0167 (11)	0.0148 (11)	0.0181 (11)	−0.0024 (9)	−0.0047 (9)	0.0042 (9)
C21	0.0183 (11)	0.0140 (11)	0.0159 (11)	0.0009 (9)	0.0004 (9)	−0.0001 (8)
N1	0.0159 (9)	0.0147 (9)	0.0146 (9)	−0.0004 (7)	−0.0026 (7)	0.0027 (7)
C25	0.0210 (12)	0.0180 (11)	0.0113 (10)	−0.0003 (9)	−0.0023 (9)	−0.0005 (8)
C18	0.0176 (12)	0.0190 (12)	0.0215 (12)	0.0000 (9)	0.0028 (10)	0.0010 (9)
C17	0.0142 (11)	0.0170 (11)	0.0119 (10)	0.0008 (9)	−0.0032 (9)	0.0021 (8)
N3	0.0176 (10)	0.0143 (9)	0.0192 (10)	0.0010 (7)	0.0000 (8)	0.0007 (7)
C19	0.0145 (11)	0.0140 (10)	0.0130 (10)	0.0019 (8)	−0.0019 (9)	0.0026 (8)
N2	0.0163 (9)	0.0153 (9)	0.0156 (9)	−0.0018 (7)	−0.0031 (8)	0.0035 (7)
C28	0.0192 (12)	0.0163 (11)	0.0194 (12)	0.0006 (9)	−0.0012 (9)	0.0022 (9)
C24	0.0176 (12)	0.0169 (11)	0.0159 (11)	0.0026 (9)	0.0013 (9)	0.0009 (9)
C20	0.0162 (11)	0.0128 (10)	0.0155 (11)	−0.0024 (9)	−0.0017 (9)	0.0044 (8)
C26	0.0261 (13)	0.0248 (12)	0.0197 (12)	−0.0017 (10)	−0.0091 (10)	0.0001 (10)
N4	0.0189 (10)	0.0123 (9)	0.0205 (10)	−0.0023 (8)	0.0008 (8)	0.0015 (8)
C29	0.0183 (12)	0.0174 (11)	0.0185 (11)	0.0037 (9)	−0.0019 (9)	0.0010 (9)
C5	0.0219 (12)	0.0259 (13)	0.0189 (12)	−0.0036 (10)	−0.0074 (10)	0.0005 (10)
C27	0.0231 (12)	0.0176 (11)	0.0206 (12)	−0.0025 (9)	−0.0021 (10)	−0.0011 (9)
C13	0.0182 (12)	0.0160 (11)	0.0221 (12)	−0.0039 (9)	−0.0002 (10)	0.0039 (9)
C4	0.0311 (14)	0.0244 (13)	0.0240 (13)	−0.0004 (11)	−0.0097 (11)	−0.0013 (10)
C2	0.0265 (13)	0.0285 (13)	0.0200 (12)	−0.0027 (10)	−0.0053 (10)	0.0057 (10)
C3	0.0222 (13)	0.0265 (13)	0.0227 (12)	0.0033 (10)	−0.0073 (10)	0.0050 (10)
C7	0.0218 (14)	0.0541 (18)	0.0310 (14)	−0.0112 (12)	−0.0094 (11)	0.0103 (13)
C30	0.0319 (14)	0.0211 (13)	0.0310 (14)	0.0023 (11)	0.0025 (11)	0.0002 (10)
C16	0.0268 (13)	0.0197 (12)	0.0246 (13)	−0.0052 (10)	−0.0008 (10)	0.0012 (10)
C9	0.0209 (12)	0.0143 (11)	0.0217 (12)	0.0032 (9)	0.0043 (10)	0.0009 (9)
C1	0.0185 (12)	0.0177 (11)	0.0170 (11)	−0.0011 (9)	−0.0029 (9)	0.0013 (9)
C31	0.0246 (13)	0.0218 (12)	0.0213 (12)	0.0010 (10)	0.0004 (10)	0.0063 (9)
C14	0.0190 (13)	0.0215 (12)	0.0431 (16)	−0.0029 (10)	0.0051 (11)	0.0025 (11)
C15	0.0369 (15)	0.0260 (13)	0.0222 (13)	−0.0107 (11)	−0.0018 (11)	0.0051 (10)
C12	0.0398 (16)	0.0348 (15)	0.0306 (14)	0.0201 (12)	0.0108 (12)	0.0092 (12)
C6	0.0351 (15)	0.0410 (16)	0.0249 (14)	−0.0128 (12)	−0.0129 (12)	0.0138 (11)
C11	0.0410 (17)	0.0277 (14)	0.0497 (18)	0.0137 (12)	−0.0085 (14)	−0.0171 (13)
C10	0.0315 (15)	0.0234 (13)	0.0474 (17)	0.0010 (11)	0.0251 (13)	−0.0052 (12)
C8	0.0513 (18)	0.0394 (16)	0.0336 (15)	0.0051 (13)	−0.0202 (14)	−0.0126 (12)
C22	0.0257 (12)	0.0148 (11)	0.0145 (11)	−0.0028 (9)	−0.0024 (10)	−0.0015 (9)

### Geometric parameters (Å, °)

**Table:**

P5—N7	1.5463 (17)	P2—N3	1.5457 (17)
P5—N6	1.6798 (18)	P2—N2	1.6735 (18)
P5—N5	1.6810 (17)	P2—N1	1.6904 (17)
P5—C48	1.831 (2)	P2—C17	1.827 (2)
P6—O5	1.4606 (15)	P1—N4	1.6577 (19)
P6—O7	1.5799 (15)	P1—N1	1.7419 (18)
P6—O6	1.5832 (15)	P1—N2	1.7486 (17)
P6—C50	1.809 (2)	P3—O1	1.4601 (15)
P4—N8	1.6616 (19)	P3—O2	1.5826 (15)
P4—N5	1.7362 (18)	P3—O3	1.5840 (15)
P4—N6	1.7503 (17)	P3—C19	1.808 (2)
O8—C54	1.361 (2)	O4—C23	1.364 (2)
O8—C57	1.430 (3)	O4—C26	1.425 (3)
O6—C60	1.454 (2)	O2—C29	1.462 (2)
O7—C58	1.459 (2)	O3—C27	1.451 (2)
N6—C36	1.488 (3)	C23—C22	1.386 (3)
C54—C53	1.387 (3)	C23—C24	1.389 (3)
C54—C55	1.392 (3)	C21—C22	1.384 (3)
C48—C49	1.327 (3)	C21—C20	1.388 (3)
C48—C50	1.532 (3)	C21—H21	0.9300
C55—C56	1.380 (3)	N1—C1	1.486 (3)
C55—H55	0.9300	C25—C24	1.375 (3)
N7—C40	1.479 (3)	C25—C20	1.398 (3)
C51—C52	1.385 (3)	C25—H25	0.9300
C51—C56	1.396 (3)	C18—C17	1.323 (3)
C51—C50	1.526 (3)	C18—H18A	0.9300
N8—C44	1.484 (3)	C18—H18B	0.9300
N8—H8D	0.82 (2)	C17—C19	1.530 (3)
C56—H56	0.9300	N3—C9	1.478 (3)
C58—C59	1.521 (3)	C19—C20	1.523 (3)
C58—H58A	0.9700	C19—H19	0.9800
C58—H58B	0.9700	N2—C5	1.485 (3)
C35—C32	1.517 (3)	C28—C29	1.523 (3)
C35—H35A	0.9600	C28—C31	1.524 (3)
C35—H35B	0.9600	C28—C27	1.526 (3)
C35—H35C	0.9600	C28—C30	1.531 (3)
C52—C53	1.390 (3)	C24—H24	0.9300
C52—H52	0.9300	C26—H26A	0.9600
C36—C39	1.521 (3)	C26—H26B	0.9600
C36—C37	1.525 (3)	C26—H26C	0.9600
C36—C38	1.528 (3)	N4—C13	1.481 (3)
C49—H49A	0.9300	N4—H4D	0.84 (3)
C49—H49B	0.9300	C29—H29A	0.9700
C57—H57A	0.9600	C29—H29B	0.9700
C57—H57B	0.9600	C5—C7	1.524 (3)
C57—H57C	0.9600	C5—C6	1.527 (3)
C53—H53	0.9300	C5—C8	1.527 (3)
C32—N5	1.483 (3)	C27—H27A	0.9700
C32—C33	1.526 (3)	C27—H27B	0.9700
C32—C34	1.527 (3)	C13—C16	1.522 (3)
C40—C42	1.523 (3)	C13—C14	1.526 (3)
C40—C43	1.530 (3)	C13—C15	1.528 (3)
C40—C41	1.531 (3)	C4—C1	1.521 (3)
C59—C60	1.524 (3)	C4—H4A	0.9600
C59—C62	1.525 (3)	C4—H4B	0.9600
C59—C61	1.532 (3)	C4—H4C	0.9600
C50—H50	0.9800	C2—C1	1.528 (3)
C34—H34A	0.9600	C2—H2A	0.9600
C34—H34B	0.9600	C2—H2B	0.9600
C34—H34C	0.9600	C2—H2C	0.9600
C60—H60A	0.9700	C3—C1	1.532 (3)
C60—H60B	0.9700	C3—H3A	0.9600
C33—H33A	0.9600	C3—H3B	0.9600
C33—H33B	0.9600	C3—H3C	0.9600
C33—H33C	0.9600	C7—H7A	0.9600
C61—H61A	0.9600	C7—H7B	0.9600
C61—H61B	0.9600	C7—H7C	0.9600
C61—H61C	0.9600	C30—H30A	0.9600
C44—C47	1.519 (3)	C30—H30B	0.9600
C44—C46	1.527 (3)	C30—H30C	0.9600
C44—C45	1.530 (3)	C16—H16A	0.9600
C45—H45A	0.9600	C16—H16B	0.9600
C45—H45B	0.9600	C16—H16C	0.9600
C45—H45C	0.9600	C9—C10	1.515 (3)
C43—H43A	0.9600	C9—C11	1.521 (3)
C43—H43B	0.9600	C9—C12	1.523 (3)
C43—H43C	0.9600	C31—H31A	0.9600
C37—H37A	0.9600	C31—H31B	0.9600
C37—H37B	0.9600	C31—H31C	0.9600
C37—H37C	0.9600	C14—H14A	0.9600
C39—H39A	0.9600	C14—H14B	0.9600
C39—H39B	0.9600	C14—H14C	0.9600
C39—H39C	0.9600	C15—H15A	0.9600
C62—H62A	0.9600	C15—H15B	0.9600
C62—H62B	0.9600	C15—H15C	0.9600
C62—H62C	0.9600	C12—H12A	0.9600
C41—H41A	0.9600	C12—H12B	0.9600
C41—H41B	0.9600	C12—H12C	0.9600
C41—H41C	0.9600	C6—H6A	0.9600
C38—H38A	0.9600	C6—H6B	0.9600
C38—H38B	0.9600	C6—H6C	0.9600
C38—H38C	0.9600	C11—H11A	0.9600
C42—H42A	0.9600	C11—H11B	0.9600
C42—H42B	0.9600	C11—H11C	0.9600
C42—H42C	0.9600	C10—H10A	0.9600
C47—H47A	0.9600	C10—H10B	0.9600
C47—H47B	0.9600	C10—H10C	0.9600
C47—H47C	0.9600	C8—H8A	0.9600
C46—H46A	0.9600	C8—H8B	0.9600
C46—H46B	0.9600	C8—H8C	0.9600
C46—H46C	0.9600	C22—H22	0.9300
			
N7—P5—N6	124.35 (9)	N3—P2—N2	124.76 (9)
N7—P5—N5	124.74 (9)	N3—P2—N1	124.18 (9)
N6—P5—N5	83.15 (9)	N2—P2—N1	83.44 (9)
N7—P5—C48	104.57 (9)	N3—P2—C17	104.18 (9)
N6—P5—C48	110.55 (9)	N2—P2—C17	109.04 (9)
N5—P5—C48	107.94 (9)	N1—P2—C17	109.89 (9)
O5—P6—O7	112.08 (8)	N4—P1—N1	104.25 (9)
O5—P6—O6	112.22 (8)	N4—P1—N2	107.84 (9)
O7—P6—O6	104.38 (8)	N1—P1—N2	79.78 (8)
O5—P6—C50	114.40 (9)	O1—P3—O2	111.94 (8)
O7—P6—C50	106.75 (9)	O1—P3—O3	111.96 (8)
O6—P6—C50	106.31 (9)	O2—P3—O3	104.51 (8)
N8—P4—N5	105.48 (9)	O1—P3—C19	115.86 (9)
N8—P4—N6	108.74 (9)	O2—P3—C19	105.64 (9)
N5—P4—N6	79.54 (8)	O3—P3—C19	106.07 (9)
C54—O8—C57	116.51 (17)	C23—O4—C26	117.37 (17)
C60—O6—P6	119.34 (13)	C29—O2—P3	117.98 (13)
C58—O7—P6	118.72 (13)	C27—O3—P3	118.86 (13)
C36—N6—P5	132.78 (14)	O4—C23—C22	125.48 (19)
C36—N6—P4	126.17 (14)	O4—C23—C24	115.15 (18)
P5—N6—P4	97.72 (9)	C22—C23—C24	119.4 (2)
O8—C54—C53	124.7 (2)	C22—C21—C20	121.72 (19)
O8—C54—C55	115.91 (19)	C22—C21—H21	119.1
C53—C54—C55	119.35 (19)	C20—C21—H21	119.1
C49—C48—C50	121.78 (19)	C1—N1—P2	131.48 (14)
C49—C48—P5	117.78 (16)	C1—N1—P1	126.07 (14)
C50—C48—P5	120.34 (14)	P2—N1—P1	97.61 (9)
C56—C55—C54	120.59 (19)	C24—C25—C20	120.8 (2)
C56—C55—H55	119.7	C24—C25—H25	119.6
C54—C55—H55	119.7	C20—C25—H25	119.6
C40—N7—P5	128.33 (15)	C17—C18—H18A	120.0
C52—C51—C56	117.58 (19)	C17—C18—H18B	120.0
C52—C51—C50	119.41 (18)	H18A—C18—H18B	120.0
C56—C51—C50	122.96 (19)	C18—C17—C19	122.28 (19)
C44—N8—P4	125.36 (15)	C18—C17—P2	118.82 (17)
C44—N8—H8D	111.8 (17)	C19—C17—P2	118.84 (14)
P4—N8—H8D	119.6 (17)	C9—N3—P2	129.59 (15)
C55—C56—C51	121.0 (2)	C20—C19—C17	113.54 (16)
C55—C56—H56	119.5	C20—C19—P3	111.75 (14)
C51—C56—H56	119.5	C17—C19—P3	111.47 (14)
O7—C58—C59	111.41 (16)	C20—C19—H19	106.5
O7—C58—H58A	109.3	C17—C19—H19	106.5
C59—C58—H58A	109.3	P3—C19—H19	106.5
O7—C58—H58B	109.3	C5—N2—P2	133.49 (14)
C59—C58—H58B	109.3	C5—N2—P1	126.78 (14)
H58A—C58—H58B	108.0	P2—N2—P1	97.99 (9)
C32—C35—H35A	109.5	C29—C28—C31	110.21 (18)
C32—C35—H35B	109.5	C29—C28—C27	109.06 (17)
H35A—C35—H35B	109.5	C31—C28—C27	111.21 (18)
C32—C35—H35C	109.5	C29—C28—C30	108.57 (18)
H35A—C35—H35C	109.5	C31—C28—C30	110.11 (18)
H35B—C35—H35C	109.5	C27—C28—C30	107.60 (18)
C51—C52—C53	122.26 (19)	C25—C24—C23	120.63 (19)
C51—C52—H52	118.9	C25—C24—H24	119.7
C53—C52—H52	118.9	C23—C24—H24	119.7
N6—C36—C39	109.13 (17)	C21—C20—C25	117.83 (19)
N6—C36—C37	108.16 (18)	C21—C20—C19	119.76 (18)
C39—C36—C37	109.28 (19)	C25—C20—C19	122.39 (19)
N6—C36—C38	111.29 (18)	O4—C26—H26A	109.5
C39—C36—C38	109.10 (19)	O4—C26—H26B	109.5
C37—C36—C38	109.9 (2)	H26A—C26—H26B	109.5
C48—C49—H49A	120.0	O4—C26—H26C	109.5
C48—C49—H49B	120.0	H26A—C26—H26C	109.5
H49A—C49—H49B	120.0	H26B—C26—H26C	109.5
O8—C57—H57A	109.5	C13—N4—P1	126.02 (15)
O8—C57—H57B	109.5	C13—N4—H4D	113.2 (18)
H57A—C57—H57B	109.5	P1—N4—H4D	119.7 (18)
O8—C57—H57C	109.5	O2—C29—C28	110.96 (16)
H57A—C57—H57C	109.5	O2—C29—H29A	109.4
H57B—C57—H57C	109.5	C28—C29—H29A	109.4
C54—C53—C52	119.2 (2)	O2—C29—H29B	109.4
C54—C53—H53	120.4	C28—C29—H29B	109.4
C52—C53—H53	120.4	H29A—C29—H29B	108.0
N5—C32—C35	110.04 (17)	N2—C5—C7	109.23 (18)
N5—C32—C33	109.30 (17)	N2—C5—C6	109.41 (18)
C35—C32—C33	109.83 (18)	C7—C5—C6	109.3 (2)
N5—C32—C34	109.50 (17)	N2—C5—C8	110.65 (18)
C35—C32—C34	108.71 (18)	C7—C5—C8	109.4 (2)
C33—C32—C34	109.44 (18)	C6—C5—C8	108.9 (2)
N7—C40—C42	114.31 (17)	O3—C27—C28	112.45 (17)
N7—C40—C43	108.27 (17)	O3—C27—H27A	109.1
C42—C40—C43	108.48 (19)	C28—C27—H27A	109.1
N7—C40—C41	108.59 (17)	O3—C27—H27B	109.1
C42—C40—C41	108.72 (18)	C28—C27—H27B	109.1
C43—C40—C41	108.32 (18)	H27A—C27—H27B	107.8
C58—C59—C60	108.60 (17)	N4—C13—C16	110.99 (18)
C58—C59—C62	110.83 (18)	N4—C13—C14	107.38 (17)
C60—C59—C62	111.06 (18)	C16—C13—C14	109.91 (19)
C58—C59—C61	108.43 (18)	N4—C13—C15	110.10 (18)
C60—C59—C61	107.80 (18)	C16—C13—C15	109.44 (18)
C62—C59—C61	110.02 (18)	C14—C13—C15	108.99 (19)
C32—N5—P5	132.65 (14)	C1—C4—H4A	109.5
C32—N5—P4	127.28 (14)	C1—C4—H4B	109.5
P5—N5—P4	98.22 (9)	H4A—C4—H4B	109.5
C51—C50—C48	113.51 (16)	C1—C4—H4C	109.5
C51—C50—P6	110.54 (14)	H4A—C4—H4C	109.5
C48—C50—P6	110.95 (14)	H4B—C4—H4C	109.5
C51—C50—H50	107.2	C1—C2—H2A	109.5
C48—C50—H50	107.2	C1—C2—H2B	109.5
P6—C50—H50	107.2	H2A—C2—H2B	109.5
C32—C34—H34A	109.5	C1—C2—H2C	109.5
C32—C34—H34B	109.5	H2A—C2—H2C	109.5
H34A—C34—H34B	109.5	H2B—C2—H2C	109.5
C32—C34—H34C	109.5	C1—C3—H3A	109.5
H34A—C34—H34C	109.5	C1—C3—H3B	109.5
H34B—C34—H34C	109.5	H3A—C3—H3B	109.5
O6—C60—C59	112.22 (17)	C1—C3—H3C	109.5
O6—C60—H60A	109.2	H3A—C3—H3C	109.5
C59—C60—H60A	109.2	H3B—C3—H3C	109.5
O6—C60—H60B	109.2	C5—C7—H7A	109.5
C59—C60—H60B	109.2	C5—C7—H7B	109.5
H60A—C60—H60B	107.9	H7A—C7—H7B	109.5
C32—C33—H33A	109.5	C5—C7—H7C	109.5
C32—C33—H33B	109.5	H7A—C7—H7C	109.5
H33A—C33—H33B	109.5	H7B—C7—H7C	109.5
C32—C33—H33C	109.5	C28—C30—H30A	109.5
H33A—C33—H33C	109.5	C28—C30—H30B	109.5
H33B—C33—H33C	109.5	H30A—C30—H30B	109.5
C59—C61—H61A	109.5	C28—C30—H30C	109.5
C59—C61—H61B	109.5	H30A—C30—H30C	109.5
H61A—C61—H61B	109.5	H30B—C30—H30C	109.5
C59—C61—H61C	109.5	C13—C16—H16A	109.5
H61A—C61—H61C	109.5	C13—C16—H16B	109.5
H61B—C61—H61C	109.5	H16A—C16—H16B	109.5
N8—C44—C47	111.24 (18)	C13—C16—H16C	109.5
N8—C44—C46	107.63 (17)	H16A—C16—H16C	109.5
C47—C44—C46	109.83 (19)	H16B—C16—H16C	109.5
N8—C44—C45	109.44 (18)	N3—C9—C10	113.80 (18)
C47—C44—C45	109.77 (19)	N3—C9—C11	109.23 (19)
C46—C44—C45	108.88 (19)	C10—C9—C11	109.1 (2)
C44—C45—H45A	109.5	N3—C9—C12	108.13 (17)
C44—C45—H45B	109.5	C10—C9—C12	108.2 (2)
H45A—C45—H45B	109.5	C11—C9—C12	108.2 (2)
C44—C45—H45C	109.5	N1—C1—C4	109.14 (17)
H45A—C45—H45C	109.5	N1—C1—C2	108.54 (17)
H45B—C45—H45C	109.5	C4—C1—C2	108.54 (18)
C40—C43—H43A	109.5	N1—C1—C3	112.22 (17)
C40—C43—H43B	109.5	C4—C1—C3	109.96 (19)
H43A—C43—H43B	109.5	C2—C1—C3	108.35 (18)
C40—C43—H43C	109.5	C28—C31—H31A	109.5
H43A—C43—H43C	109.5	C28—C31—H31B	109.5
H43B—C43—H43C	109.5	H31A—C31—H31B	109.5
C36—C37—H37A	109.5	C28—C31—H31C	109.5
C36—C37—H37B	109.5	H31A—C31—H31C	109.5
H37A—C37—H37B	109.5	H31B—C31—H31C	109.5
C36—C37—H37C	109.5	C13—C14—H14A	109.5
H37A—C37—H37C	109.5	C13—C14—H14B	109.5
H37B—C37—H37C	109.5	H14A—C14—H14B	109.5
C36—C39—H39A	109.5	C13—C14—H14C	109.5
C36—C39—H39B	109.5	H14A—C14—H14C	109.5
H39A—C39—H39B	109.5	H14B—C14—H14C	109.5
C36—C39—H39C	109.5	C13—C15—H15A	109.5
H39A—C39—H39C	109.5	C13—C15—H15B	109.5
H39B—C39—H39C	109.5	H15A—C15—H15B	109.5
C59—C62—H62A	109.5	C13—C15—H15C	109.5
C59—C62—H62B	109.5	H15A—C15—H15C	109.5
H62A—C62—H62B	109.5	H15B—C15—H15C	109.5
C59—C62—H62C	109.5	C9—C12—H12A	109.5
H62A—C62—H62C	109.5	C9—C12—H12B	109.5
H62B—C62—H62C	109.5	H12A—C12—H12B	109.5
C40—C41—H41A	109.5	C9—C12—H12C	109.5
C40—C41—H41B	109.5	H12A—C12—H12C	109.5
H41A—C41—H41B	109.5	H12B—C12—H12C	109.5
C40—C41—H41C	109.5	C5—C6—H6A	109.5
H41A—C41—H41C	109.5	C5—C6—H6B	109.5
H41B—C41—H41C	109.5	H6A—C6—H6B	109.5
C36—C38—H38A	109.5	C5—C6—H6C	109.5
C36—C38—H38B	109.5	H6A—C6—H6C	109.5
H38A—C38—H38B	109.5	H6B—C6—H6C	109.5
C36—C38—H38C	109.5	C9—C11—H11A	109.5
H38A—C38—H38C	109.5	C9—C11—H11B	109.5
H38B—C38—H38C	109.5	H11A—C11—H11B	109.5
C40—C42—H42A	109.5	C9—C11—H11C	109.5
C40—C42—H42B	109.5	H11A—C11—H11C	109.5
H42A—C42—H42B	109.5	H11B—C11—H11C	109.5
C40—C42—H42C	109.5	C9—C10—H10A	109.5
H42A—C42—H42C	109.5	C9—C10—H10B	109.5
H42B—C42—H42C	109.5	H10A—C10—H10B	109.5
C44—C47—H47A	109.5	C9—C10—H10C	109.5
C44—C47—H47B	109.5	H10A—C10—H10C	109.5
H47A—C47—H47B	109.5	H10B—C10—H10C	109.5
C44—C47—H47C	109.5	C5—C8—H8A	109.5
H47A—C47—H47C	109.5	C5—C8—H8B	109.5
H47B—C47—H47C	109.5	H8A—C8—H8B	109.5
C44—C46—H46A	109.5	C5—C8—H8C	109.5
C44—C46—H46B	109.5	H8A—C8—H8C	109.5
H46A—C46—H46B	109.5	H8B—C8—H8C	109.5
C44—C46—H46C	109.5	C21—C22—C23	119.6 (2)
H46A—C46—H46C	109.5	C21—C22—H22	120.2
H46B—C46—H46C	109.5	C23—C22—H22	120.2

### Hydrogen-bond geometry (Å, °)

**Table:**

*D*—H···*A*	*D*—H	H···*A*	*D*···*A*	*D*—H···*A*
N8—H8D···O1^i^	0.82 (2)	2.61 (3)	3.394 (2)	160 (2)
N4—H4D···O5^ii^	0.84 (3)	2.58 (3)	3.378 (2)	158 (2)
C26—H26B···O5^iii^	0.96	2.49	3.325 (3)	145

Symmetry codes: (i) −*x*+2, −*y*+1, −*z*; (ii) −*x*+1, −*y*+1, −*z*+1; (iii) −*x*+2, −*y*+1, −*z*+1.
